# Supplementary material for: A Likelihood Approach for Real-Time Calibration of Stochastic Compartmental Epidemic Models
Source: PLoS Comput Biol. 2017 Jan 17;13(1):e1005257. doi: 10.1371/journal.pcbi.1005257 (PMC5240920; doi:10.1371/journal.pcbi.1005257)
Supplement: S1 File — (TAR.GZ) [file pcbi.1005257.s014.tar.gz › HSPH_Online-SI-Revision/MSS10/n100-severe/n100-severe_NEW_table-4.pdf]

1	R0	{1.65278, 9.06793, 0.1}	{1.282, 3.73657, 0.2}	{{1.22544, 3.07157, 0.}, {1.3618, 4.98002, 1.}}	{22.4336, 58.7935, 260.}	inf	4882.	2002.8	{1584.53, 2774.72}	58.9758	0.397896	newly	4385.	4155.68	{3719., 4502.}	6.83101	0.0312836	Reff	1.41083	1.13099	{1.08886, 1.19429}	19.8353	ar	66 342.	40 137.6	{35 602., 48 321.}	39.4989
2	R0	{1.4321, 3.80644, 0.1}	{1.58586, 4.61236, 0.18}	{{1.47608, 3.83841, 0.}, {1.53501, 4.02511, 0.}}	{10.7369, 21.1726, 268.}	inf	1776.	2184.07	{1645.2, 2206.48}	26.1146	0.0702958	newly	4438.	4765.98	{4141., 5300.}	9.84768	0.0404205	Reff	1.34796	1.48228	{1.38633, 1.4454}	9.96414	ar	54 217.	60 541.1	{56 065., 61 104.}	11.6643
3	R0	{1.68388, 4.76028, 0.1}	{2.00379, 6.90539, 1.23}	{{1.56331, 4.02511, 0.}, {2.79531, 12.1249, 2.}}	{21.1396, 50.3545, 1150.}	inf	2116.	3163.35	{1882.34, 5716.48}	54.226	0.162932	newly	4947.	4952.	{4544., 5253.}	5.16192	0.0223446	Reff	1.59182	1.86726	{1.48076, 2.54452}	19.3539	ar	68 299.	76 387.7	{62 043., 92 517.}	14.6816
4	R0	{1.66014, 6.95383, 1.}	{1.24751, 2.5364, 0.}	{{1.24751, 2.5364, 0.}, {1.24751, 2.5364, 0.}}	{24.855, 63.5251, 100.}	inf	3294.	1190.03	{964.935, 1423.55}	63.8729	0.446542	newly	4326.	4032.63	{3304., 4730.}	11.844	0.0550559	Reff	1.51479	1.16454	{1.16151, 1.16727}	23.1222	ar	66 820.	37 045.8	{35 853., 38 387.}	44.5589
5	R0	{1.69984, 11.2038, 1.}	{1.26283, 3.56186, 0.04}	{{1.22544, 3.07157, 0.}, {1.28596, 3.98513, 0.}}	{25.709, 68.2084, 100.}	inf	6557.	2064.5	{1665.55, 2437.82}	68.5146	0.506669	newly	4410.	4333.56	{3890., 4757.}	6.24444	0.0277897	Reff	1.38564	1.08608	{1.0572, 1.10453}	21.6193	ar	68 462.	38 561.6	{34 699., 41 934.}	43.6744
6	R0	{1.65876, 6.66217, 1.}	{1.28808, 2.83148, 0.}	{{1.24751, 2.5364, 0.}, {1.44762, 4.00571, 0.}}	{22.3468, 57.4991, 100.}	inf	2507.	1046.79	{748.001, 1495.87}	58.2451	0.39521	newly	3575.	3350.22	{2591., 4084.}	14.659	0.0685268	Reff	1.55026	1.22245	{1.18265, 1.36799}	21.1456	ar	67 105.	40 491.2	{35 647., 54 278.}	39.6599
7	R0	{1.69966, 5.89996, 0.1}	{2.47989, 12.0408, 2.08}	{{1.47608, 4.06661, 0.}, {2.99911, 15.6207, 3.}}	{49.5609, 113.439, 2022.}	inf	2040.	4387.3	{1458.3, 5838.05}	124.224	0.33808	newly	3565.	3686.57	{3431., 3962.}	5.83366	0.0248278	Reff	1.61145	2.28518	{1.40964, 2.72047}	45.3004	ar	69 396.	85 175.9	{56 792., 93 890.}	27.8628
8	R0	{1.53428, 6.3734, 0.1}	{1.31994, 3.93956, 0.92}	{{1.25474, 3.26445, 0.}, {1.41571, 4.94504, 2.}}	{13.9702, 38.1875, 932.}	inf	2925.	1750.76	{1248.96, 2314.93}	40.1448	0.238007	newly	3946.	3675.99	{3132., 4115.}	8.85226	0.0416628	Reff	1.38868	1.20983	{1.15135, 1.29361}	12.8789	ar	59 855.	43 623.1	{36 537., 52 673.}	27.1187
9	R0	{1.58939, 3.48608, 0.1}	{2.22893, 9.06113, 0.46}	{{1.737, 5.43578, 0.}, {2.71225, 12.7047, 1.}}	{40.2383, 159.923, 476.}	inf	458.	1237.91	{759.137, 1742.49}	170.286	0.412534	newly	1645.	1552.68	{1399., 1689.}	7.52097	0.0342196	Reff	1.56577	2.17714	{1.70647, 2.63929}	39.0455	ar	62 575.	82 920.7	{70 798., 91 682.}	32.514
10	R0	{1.58528, 5.5593, 0.1}	{1.28266, 2.79312, 0.03}	{{1.24751, 2.5364, 0.}, {1.44762, 4.00571, 0.}}	{19.0894, 49.7578, 128.}	inf	2476.	1314.4	{911.964, 1843.54}	46.9143	0.289758	newly	4136.	4119.43	{3098., 4986.}	13.3083	0.0602454	Reff	1.4648	1.19987	{1.16474, 1.34583}	18.0864	ar	63 039.	40 307.	{35 889., 54 252.}	36.0602
11	R0	{1.58305, 4.62721, 0.1}	{1.34443, 1.83775, 1.44}	{{1.2663, 1.35135, 0.}, {1.39162, 1.91565, 4.}}	{15.0732, 60.2838, 1462.}	inf	1827.	748.216	{324.453, 1129.35}	59.0467	0.434543	newly	3948.	4839.96	{2283., 7456.}	42.2016	0.166274	Reff	1.50095	1.2892	{1.21548, 1.33896}	14.1076	ar	63 222.	45 543.3	{38 130., 50 729.}	27.9628
12	R0	{1.70234, 9.34626, 0.1}	{1.25218, 3.19998, 0.}	{{1.24751, 3.07157, 0.}, {1.25474, 3.26445, 0.}}	{26.4435, 65.7619, 100.}	inf	5054.	1708.17	{1435.97, 1910.07}	66.2017	0.474181	newly	4443.	4181.59	{3718., 4539.}	7.42854	0.0345313	Reff	1.46651	1.12061	{1.11894, 1.1246}	23.5868	ar	68 213.	37 627.3	{35 821., 38 986.}	44.8385
13	R0	{1.68556, 9.74954, 1.}	{1.24828, 2.69471, 0.03}	{{1.24751, 2.5364, 0.}, {1.25474, 3.26445, 0.}}	{25.9427, 72.3607, 101.}	inf	5632.	1557.94	{1208.15, 1903.}	72.3377	0.56507	newly	4572.	4482.87	{3741., 5257.}	10.243	0.0459249	Reff	1.41078	1.09563	{1.09302, 1.09977}	22.3384	ar	68 426.	36 129.4	{34 537., 38 013.}	47.1993
14	R0	{1.59876, 9.78897, 1.}	{1.22573, 3.0735, 0.}	{{1.22544, 3.07157, 0.}, {1.22544, 3.07157, 0.}}	{23.3325, 68.6024, 100.}	inf	4188.	1296.43	{1093.96, 1483.55}	69.0442	0.512495	newly	3466.	3277.24	{2714., 3727.}	10.2089	0.0473713	Reff	1.39393	1.10413	{1.10127, 1.10635}	20.7898	ar	63 553.	34 045.8	{32 396., 35 362.}	46.4293
15	R0	{1.43577, 5.12819, 1.}	{1.2415, 3.0616, 0.}	{{1.22544, 2.5364, 0.}, {1.25474, 3.26445, 0.}}	{13.5309, 40.2986, 100.}	inf	1915.	1043.64	{772.225, 1290.42}	45.5021	0.272213	newly	3233.	2846.66	{2277., 3428.}	15.4865	0.0753163	Reff	1.33374	1.16409	{1.14776, 1.17703}	12.72	ar	53 259.	36 149.5	{34 105., 38 333.}	32.1252
16	R0	{1.58866, 8.73191, 1.}	{1.23976, 3.16936, 0.}	{{1.22544, 3.07157, 0.}, {1.25474, 3.26445, 0.}}	{21.9618, 63.7037, 100.}	inf	3460.	1223.86	{1019.28, 1408.3}	64.6284	0.456021	newly	3246.	3158.07	{2587., 3621.}	9.87708	0.0448996	Reff	1.43615	1.14846	{1.13358, 1.16367}	20.032	ar	64 349.	36 226.7	{33 890., 38 404.}	43.7027
17	R0	{1.43012, 3.8163, 0.1}	{1.27762, 2.83213, 0.08}	{{1.24751, 2.5364, 0.}, {1.24751, 2.5364, 0.}}	{14.3629, 39.9452, 176.}	inf	970.	654.041	{367.765, 808.305}	47.5366	0.253982	newly	2605.	2104.02	{1350., 2819.}	23.1209	0.12423	Reff	1.37832	1.23419	{1.20453, 1.20994}	13.8663	ar	53 353.	37 653.9	{35 204., 37 984.}	32.348
18	R0	{1.47965, 5.16472, 0.1}	{1.24751, 2.5364, 0.}	{{1.24751, 2.5364, 0.}, {1.24751, 2.5364, 0.}}	{15.6886, 50.8898, 100.}	inf	2352.	1122.44	{955.35, 1298.52}	52.2774	0.325212	newly	4058.	3798.02	{3204., 4470.}	11.2346	0.0524493	Reff	1.36212	1.16376	{1.16155, 1.16582}	14.5625	ar	56 086.	36 685.2	{35 301., 37 700.}	34.5912
19	R0	{1.57191, 5.2314, 0.1}	{1.44251, 3.97014, 0.03}	{{1.44762, 4.00571, 0.}, {1.44762, 4.00571, 0.}}	{8.23192, 24.1095, 128.}	inf	3247.	2460.74	{2218.15, 2717.56}	24.2151	0.124131	newly	5657.	5569.62	{5019., 6117.}	6.0951	0.0273425	Reff	1.41239	1.30739	{1.30787, 1.31546}	7.43432	ar	63 119.	54 040.4	{53 552., 55 371.}	14.3833
20	R0	{1.54435, 5.32911, 0.1}	{1.94493, 9.68342, 1.}	{{1.95791, 9.82151, 1.}, {1.95791, 9.82151, 1.}}	{26.5194, 83.4486, 904.}	inf	2562.	4442.49	{4342.79, 4654.49}	75.4966	0.249563	newly	4511.	4186.36	{4025., 4377.}	7.2924	0.034791	Reff	1.42398	1.75634	{1.76363, 1.77029}	23.8737	ar	61 870.	77 762.8	{78 102., 78 866.}	26.9817
21	R0	{1.57932, 4.90031, 1.}	{2.94862, 15.3182, 2.1}	{{2.63407, 13.6136, 2.}, {2.99911, 15.6207, 2.}}	{86.7021, 212.596, 110.}	inf	1638.	5800.82	{4961.9, 6078.54}	254.14	0.54828	newly	3321.	3898.01	{3668., 4064.}	17.3746	0.0692819	Reff	1.50468	2.68616	{2.42787, 2.73136}	78.5202	ar	63 595.	93 648.2	{91 202., 94 186.}	47.2572
22	R0	{1.47044, 4.07694, 1.}	{2.22369, 13.3004, 2.28}	{{1.24751, 2.5364, 0.}, {2.85956, 19.8999, 3.}}	{58.5331, 244.109, 176.}	inf	941.	3170.72	{457.601, 4812.63}	257.45	0.538682	newly	2369.	2172.25	{1696., 2360.}	10.5509	0.0528047	Reff	1.4165	2.08367	{1.20576, 2.64316}	54.237	ar	55 895.	76 620.5	{35 887., 92 932.}	53.9201
23	R0	{1.64997, 3.34546, 0.1}	{2.0465, 6.74348, 0.23}	{{1.98388, 6.25659, 0.}, {2.23156, 8.18891, 1.}}	{24.5663, 102.013, 304.}	inf	758.	1469.6	{1153.32, 1672.74}	95.0319	0.280968	newly	3196.	2630.11	{2289., 2953.}	17.9797	0.0884634	Reff	1.61331	1.98552	{1.9246, 2.162}	23.6004	ar	66 404.	79 833.	{78 746., 85 152.}	20.9247
24	R0	{1.67405, 4.50254, 0.1}	{1.52568, 3.89633, 0.19}	{{1.47608, 3.72349, 0.}, {1.53501, 4.02511, 1.}}	{9.03431, 13.9869, 264.}	inf	1073.	778.509	{571.422, 933.139}	27.6377	0.149409	newly	2825.	2242.74	{1697., 2705.}	22.1529	0.113201	Reff	1.63156	1.49142	{1.44221, 1.50362}	8.76071	ar	68 017.	59 797.5	{56 121., 61 548.}	12.2704
25	R0	{1.50663, 3.1286, 0.1}	{1.48102, 2.86262, 1.04}	{{1.39162, 1.89883, 0.}, {1.64953, 3.72349, 2.}}	{5.26191, 18.6381, 980.}	inf	639.	597.648	{358.649, 836.873}	25.1206	0.136747	newly	2373.	2670.21	{1671., 3860.}	30.6696	0.13339	Reff	1.47669	1.45212	{1.36942, 1.61351}	5.16384	ar	58 823.	56 434.	{50 664., 66 565.}	9.06802
26	R0	{1.69822, 6.34992, 0.1}	{1.25179, 2.5664, 0.}	{{1.24751, 2.5364, 0.}, {1.24751, 2.5364, 0.}}	{26.2882, 59.5837, 100.}	inf	2762.	1122.98	{924.245, 1326.66}	59.3419	0.39582	newly	4173.	3846.99	{3080., 4530.}	12.1896	0.0574423	Reff	1.57542	1.18177	{1.17531, 1.18024}	24.9873	ar	69 816.	37 653.3	{36 076., 38 672.}	46.0678
27	R0	{1.55869, 4.92988, 0.1}	{1.67949, 5.79678, 0.22}	{{1.44762, 3.83841, 0.}, {2.4534, 12.3139, 1.}}	{16.8668, 46.7161, 280.}	inf	1917.	2410.04	{1463.38, 5174.9}	48.0562	0.148732	newly	3834.	4048.96	{3600., 4437.}	8.41523	0.0349559	Reff	1.46882	1.5686	{1.36838, 2.22844}	15.492	ar	62 344.	63 236.1	{54 891., 88 585.}	16.9146
28	R0	{1.46085, 6.06899, 0.1}	{1.25548, 3.22108, 0.02}	{{1.25474, 3.26445, 0.}, {1.25474, 3.26445, 0.}}	{14.0584, 46.9256, 118.}	inf	3200.	1715.26	{1442.53, 1928.7}	46.398	0.274347	newly	4410.	4225.65	{3682., 4740.}	8.33583	0.0380205	Reff	1.29135	1.12843	{1.12457, 1.13039}	12.6163	ar	55 484.	38 083.9	{36 476., 39 308.}	31.3605
29	R0	{1.63017, 6.25164, 0.1}	{2.00346, 9.53915, 1.75}	{{1.44762, 4.00571, 0.}, {2.49952, 14.4455, 3.}}	{28.3667, 69.7953, 1694.}	inf	3520.	5250.19	{2056.86, 7984.85}	68.0898	0.230251	newly	5090.	5052.26	{4748., 5281.}	3.05226	0.0134259	Reff	1.47729	1.77179	{1.33242, 2.151}	24.7536	ar	66 231.	75 776.1	{54 153., 89 173.}	23.2238
30	R0	{1.45173, 5.68165, 0.1}	{1.52152, 6.17896, 2.24}	{{1.24751, 2.5364, 0.}, {1.70777, 8.49961, 3.}}	{10.2809, 32.909, 2166.}	inf	2741.	2862.08	{1376.56, 4014.15}	32.4294	0.147202	newly	3975.	3960.68	{3713., 4281.}	5.10038	0.0231218	Reff	1.31862	1.3785	{1.15651, 1.53205}	9.27053	ar	55 280.	57 879.9	{37 871., 69 592.}	17.086
31	R0	{1.55808, 2.77316, 0.1}	{1.44387, 2.31942, 0.3}	{{1.39162, 1.89883, 0.}, {1.65456, 3.37285, 1.}}	{9.2283, 23.9759, 342.}	inf	583.	538.834	{292.114, 799.833}	29.956	0.159743	newly	3043.	3221.91	{1895., 4892.}	31.0217	0.142517	Reff	1.52792	1.41645	{1.36385, 1.61831}	9.12661	ar	60 940.	53 894.9	{49 688., 66 646.}	14.581
32	R0	{1.56797, 3.90327, 0.1}	{1.58956, 4.36255, 0.11}	{{1.47608, 3.83841, 0.}, {1.74592, 5.58477, 0.}}	{5.86958, 18.1655, 194.}	inf	984.	1076.04	{816.674, 1395.76}	18.9201	0.0767922	newly	2775.	2764.43	{2360., 3070.}	10.8422	0.0448708	Reff	1.52355	1.54285	{1.43301, 1.68876}	5.72146	ar	62 228.	62 997.	{55 832., 71 222.}	7.85413
33	R0	{1.70027, 5.43261, 0.1}	{1.52388, 3.99823, 0.03}	{{1.47608, 3.83841, 0.}, {1.53501, 4.02511, 0.}}	{10.4606, 26.4055, 128.}	inf	2721.	1868.45	{1665.63, 2098.53}	31.3322	0.16517	newly	5236.	4775.5	{4321., 5267.}	9.78457	0.0455234	Reff	1.58887	1.43699	{1.39308, 1.45006}	9.66286	ar	68 798.	59 706.6	{56 687., 61 108.}	13.3211
34	R0	{1.61668, 6.86192, 0.1}	{1.24751, 2.5364, 0.}	{{1.24751, 2.5364, 0.}, {1.24751, 2.5364, 0.}}	{22.8349, 63.0365, 100.}	inf	3615.	1358.9	{1051.28, 1650.93}	62.4095	0.430858	newly	4645.	4468.13	{3548., 5335.}	12.4316	0.0569133	Reff	1.44922	1.14644	{1.14276, 1.15004}	20.8928	ar	64 693.	36 918.4	{35 329., 38 377.}	42.9329
35	R0	{1.55955, 3.45071, 0.1}	{2.57954, 10.9395, 2.49}	{{1.39162, 1.89883, 0.}, {2.82285, 12.7912, 3.}}	{69.0639, 232.312, 2424.}	inf	643.	1878.01	{340.361, 2306.69}	210.526	0.537764	newly	2408.	2018.96	{1779., 2133.}	24.8513	0.138553	Reff	1.53037	2.49557	{1.37205, 2.72911}	66.6224	ar	61 783.	85 478.1	{50 119., 92 876.}	44.7694
36	R0	{1.65068, 4.91307, 0.1}	{1.46617, 3.88257, 0.}	{{1.44762, 3.83841, 0.}, {1.47608, 4.06661, 0.}}	{11.1781, 20.9747, 100.}	inf	1136.	870.531	{711.703, 1042.7}	23.4284	0.12168	newly	2506.	2322.74	{1896., 2674.}	11.7606	0.0553762	Reff	1.60096	1.42586	{1.40731, 1.43754}	10.937	ar	66 453.	55 877.7	{54 622., 57 813.}	15.914
37	R0	{1.46131, 3.06714, 0.1}	{1.39259, 1.91297, 0.02}	{{1.39162, 1.89883, 0.}, {1.39162, 1.89883, 0.}}	{4.70297, 37.63, 116.}	inf	856.	588.294	{196.804, 891.277}	34.9149	0.249	newly	3280.	4178.42	{1363., 6204.}	51.7543	0.22021	Reff	1.41952	1.35648	{1.35132, 1.36135}	4.4405	ar	56 452.	50 282.8	{49 044., 51 261.}	10.9283
38	R0	{1.71034, 5.2546, 0.1}	{2.41801, 10.5557, 1.6}	{{1.737, 5.43578, 0.}, {2.59133, 11.8519, 2.}}	{42.1962, 102.757, 1540.}	inf	2107.	4083.24	{2065.82, 4727.41}	96.276	0.281127	newly	4350.	4117.16	{3900., 4289.}	5.9246	0.0266887	Reff	1.62197	2.24197	{1.64838, 2.39742}	39.0104	ar	69 816.	86 335.4	{70 586., 90 559.}	24.7158
39	R0	{1.43565, 5.57989, 1.}	{1.24844, 2.68544, 0.}	{{1.24751, 2.5364, 0.}, {1.25474, 3.26445, 0.}}	{13.0398, 51.873, 100.}	inf	2976.	1407.55	{1060.32, 1702.51}	52.7034	0.3322	newly	4327.	4291.31	{3484., 5239.}	12.7606	0.05676	Reff	1.28285	1.13515	{1.13143, 1.13947}	11.5136	ar	54 407.	36 587.3	{34 659., 38 447.}	32.7526
40	R0	{1.40327, 4.00764, 0.1}	{1.24751, 2.5364, 0.}	{{1.24751, 2.5364, 0.}, {1.24751, 2.5364, 0.}}	{11.0996, 36.7108, 100.}	inf	1552.	1027.95	{822.764, 1227.44}	33.7661	0.184201	newly	3528.	3573.86	{2807., 4208.}	12.271	0.0534754	Reff	1.32289	1.18259	{1.18004, 1.18511}	10.6054	ar	51 976.	37 159.3	{35 587., 38 626.}	28.5069
41	R0	{1.63612, 4.26741, 0.1}	{1.38709, 1.884, 0.13}	{{1.39162, 1.89883, 0.}, {1.39162, 1.89883, 0.}}	{15.2207, 55.8514, 220.}	inf	1646.	798.581	{398.797, 1223.89}	51.846	0.369505	newly	4452.	5491.11	{3000., 8236.}	40.5168	0.167023	Reff	1.56264	1.33654	{1.33378, 1.3464}	14.4692	ar	65 029.	49 690.	{48 950., 51 381.}	23.5879
42	R0	{1.57426, 6.50271, 0.1}	{2.08418, 12.0493, 1.02}	{{2.0968, 12.1899, 1.}, {2.0968, 12.1899, 1.}}	{32.3909, 85.2972, 920.}	inf	3056.	5804.09	{5681.34, 6027.84}	89.9246	0.277574	newly	4138.	4278.79	{4145., 4380.}	3.6073	0.0153241	Reff	1.43171	1.83796	{1.84411, 1.85066}	28.3753	ar	62 522.	81 746.6	{81 691., 82 528.}	30.7485
43	R0	{1.37818, 5.37636, 0.1}	{1.39976, 5.43438, 0.32}	{{1.28596, 3.98513, 0.}, {1.44485, 6.00596, 1.}}	{4.08009, 12.6405, 360.}	inf	2468.	2412.76	{1820.72, 2794.29}	11.3809	0.0526912	newly	3812.	3649.35	{3383., 3911.}	5.3628	0.0242113	Reff	1.24355	1.2636	{1.17042, 1.30251}	3.77616	ar	50 098.	50 824.7	{41 711., 55 161.}	8.64306
44	R0	{1.66606, 4.92641, 0.1}	{1.64947, 4.94175, 0.03}	{{1.47608, 3.83841, 0.}, {1.74592, 5.83416, 0.}}	{7.13304, 17.4219, 126.}	inf	1205.	1166.97	{781.06, 1439.05}	18.7169	0.0866023	newly	2554.	2555.94	{2104., 2941.}	9.92874	0.0438878	Reff	1.61485	1.59906	{1.4359, 1.69118}	6.91373	ar	67 066.	65 965.9	{56 849., 71 403.}	8.92826
45	R0	{1.623, 5.9449, 0.1}	{1.25579, 2.59579, 0.}	{{1.24751, 2.5364, 0.}, {1.24751, 2.5364, 0.}}	{22.6254, 56.3359, 100.}	inf	2171.	917.553	{670.869, 1151.74}	57.7359	0.383852	newly	3513.	3180.78	{2384., 3960.}	15.4438	0.0746801	Reff	1.52557	1.1961	{1.18554, 1.19149}	21.5963	ar	64 554.	37 612.7	{35 710., 38 349.}	41.7346
46	R0	{1.65235, 5.51234, 0.1}	{1.46027, 3.9234, 0.06}	{{1.44762, 3.83841, 0.}, {1.47608, 4.06661, 0.}}	{11.6245, 28.8251, 156.}	inf	1774.	1220.54	{1014.77, 1400.44}	31.1983	0.166135	newly	3211.	3123.38	{2634., 3501.}	8.54438	0.038645	Reff	1.57561	1.40052	{1.38671, 1.41687}	11.1131	ar	66 509.	55 439.4	{54 241., 57 206.}	16.6437
47	R0	{1.62138, 8.82208, 0.1}	{1.24715, 3.16519, 0.}	{{1.22544, 3.07157, 0.}, {1.25474, 3.26445, 0.}}	{23.0811, 64.1219, 100.}	inf	5092.	1827.65	{1576.99, 2093.8}	64.1074	0.447949	newly	4726.	4380.96	{3837., 4880.}	9.10241	0.0422411	Reff	1.36283	1.08898	{1.07057, 1.09728}	20.0945	ar	65 598.	36 888.3	{34 369., 38 482.}	43.7661
48	R0	{1.64073, 6.69261, 0.1}	{1.26952, 2.69803, 0.}	{{1.24751, 2.5364, 0.}, {1.44762, 4.00571, 0.}}	{22.6244, 59.6865, 100.}	inf	3000.	1242.97	{937.657, 1715.28}	58.5675	0.393262	newly	4095.	4025.41	{3178., 4791.}	12.5927	0.0564923	Reff	1.5074	1.18853	{1.16636, 1.34292}	21.1538	ar	66 438.	39 064.1	{35 663., 53 395.}	41.2022
49	R0	{1.65556, 3.796, 0.1}	{1.70873, 3.85835, 0.6}	{{1.39162, 1.89883, 0.}, {2.93597, 11.6547, 3.}}	{28.083, 79.8312, 658.}	inf	673.	605.779	{107.545, 1667.36}	73.2804	0.406507	newly	2370.	2309.92	{801., 4313.}	37.1114	0.193262	Reff	1.6296	1.67969	{1.37132, 2.85542}	27.4696	ar	66 575.	59 634.	{49 474., 93 568.}	27.2547
50	R0	{1.56828, 3.65713, 0.1}	{1.46028, 2.78707, 1.19}	{{1.43965, 2.60601, 1.}, {1.56331, 3.72349, 2.}}	{7.30079, 25.496, 1104.}	inf	1346.	1109.67	{733.547, 1460.59}	23.0628	0.121272	newly	4132.	4669.61	{3501., 5791.}	20.1455	0.0800945	Reff	1.50552	1.40518	{1.38056, 1.49528}	7.04062	ar	62 761.	55 283.2	{52 700., 61 561.}	12.4045
